# Outcome Comparison between Laparoscopic and Open Appendectomy: Evidence from a Nationwide Population-Based Study

**DOI:** 10.1371/journal.pone.0068662

**Published:** 2013-07-12

**Authors:** Chien-Che Wang, Chao-Chiang Tu, Pi-Chieh Wang, Herng-Ching Lin, Po-Li Wei

**Affiliations:** 1 Department of General Surgery, PoJen General Hospital, Taipei, Taiwan,; 2 Division of General Surgery, Department of Surgery, Taipei Medical University Hospital, Taipei, Taiwan; 3 Department of Family Medicine, PoJen General Hospital, Taipei, Taiwan; 4 School of Health Care Administration, Taipei Medical University, Taipei, Taiwan; 5 Department of Surgery, School of Medicine, College of Medicine, Taipei Medical University, Taipei, Taiwan; Tulane University School of Public Health and Tropical Medicine, United States of America

## Abstract

**Background:**

Mounting evidence supports the use of laparoscopic techniques for the treatment of simple appendicitis. However, most of the advantages of these techniques are of limited clinical relevance. This study compares the treatment outcomes of laparoscopic appendectomies and open appendectomies performed in Taiwan.

**Methods:**

This study uses data from the 2007 to 2009 Taiwan National Health Insurance Research Database. The study sample included 65,339 patients, hospitalized with a discharge diagnosis of acute appendicitis (33.8% underwent laparoscopic appendectomy). A generalized estimated equation (GEE) was performed to explore the relationship between the use of laparoscopy and 30-day re-admission. Hierarchical linear regressions were performed to examine the relationship between the use of laparoscopy, the length of stay (LOS), and the cost per discharge.

**Results:**

A significantly lower proportion of patients undergoing laparoscopic appendectomies were re-admitted within 30 days of their index appendectomy, in comparison to patients undergoing open appendectomies (0.66% versus 1.925, *p*<0.001). Compared with patients undergoing open appendectomies, patients undergoing laparoscopic appendectomies had a shorter LOS (4.01 versus 5.33 days, *p*<0.001) and a higher cost per discharge (NT$40,554 versus NT$38,509, *p*<0.001. In 2007, the average exchange rate was US$1 = NT$31.0). GEE revealed that the odds ratio of 30-day readmission for patients undergoing laparoscopic appendectomy was 0.38 (95% CI = 0.33–0.46) that of patients undergoing open appendectomies, after adjusting for surgeon, hospital, and patient characteristics, as well as for the clustering effect of particular surgeons and the propensity score.

**Conclusion:**

This study found that laparoscopic appendectomies had a lower 30-day re-admission rate, and a shorter LOS, but a slightly higher cost per discharge than open appendectomies.

## Introduction

Acute appendicitis is the most common emergency abdominal surgery. In 1983, Semm introduced the use of laparoscopic techniques, with the first large study of laparoscopic appendectomies reported by Pier et al. in 1991 [1], [2]. This technique allows surgeons to avoid the traditional muscle-splitting incision at the McBurney point, which was the standard treatment for over a century.

Although initially a controversial procedure, accumulating evidence supports the use of a laparoscopic appendectomy for the treatment of simple appendicitis [3]–[5]. Laparoscopic appendectomies are preferred to open surgery for simple appendicitis, but most of the advantages are of limited clinical relevance [6]. Intra-abdominal abscesses are a concern when performing laparoscopic appendectomies in cases of complicated appendicitis. A meta-analysis conducted on children with appendicitis revealed that intra-abdominal abscess formation was more common following laparoscopic surgery, although this was not statistically significant [5]. In adults, the use of laparoscopic appendectomies has been associated with a higher rate of intra-abdominal abscesses, with a consequently higher rate of interventions and re-admissions [7]. However, one study using a Nationwide Inpatient Sample database in the U.S. revealed that laparoscopic appendectomies were associated with lower morbidity, lower mortality, shorter hospital stays, and a reduction in hospital charges in adults with perforated appendicitis [8].

This study compares the treatment outcomes between laparoscopic appendectomies and open appendectomies, using a nationwide population-based dataset in Taiwan, where laparoscopic appendectomies have been in use since 1993 [9]. Although the National Health Insurance (NHI) program was founded in 1995, the reimbursement for laparoscopic appendectomies was not available until 2007. This information allowed us to compare the treatment outcomes between these two procedures.

## Methods

### Database

The dataset was sourced from the National Health Insurance Research Database (NHIRD) for the period from 2007 to 2009. Initiated in 1995, Taiwan’s NHI program is characterized by a single plan with the government as the sole insurer, comprehensive benefits, low co-payments, and free choice of healthcare providers from a widely-dispersed network. The NHI had 22.60 million members (an over 98% coverage rate of Taiwan’s 22.96 million residents) at the end of 2009. The NHIRD is published annually by the Taiwan National Health Research Institute, and contains the original claim data and registration files for all the enrollees under the NHI program. Many studies have used this dataset and have been published in international, peer-reviewed journals [10].

This study was exempted from full review by the Institutional Review Board (IRB) of the Taipei Medical University, after consulting with the director of the Taipei Medical University IRB, because the NHIRD consists of de-identified secondary data released to the public for research purposes.

### Study Sample

In total, 65,339 patients hospitalized with a discharge diagnosis of acute appendicitis (International Classification of Disease, Ninth Revision, Clinical Modification (ICD-9-CM) codes 540, 540.0, 540.1 and 540.9) between January 2007 and December 2009 were selected from the NHIRD. Of these sampled patients, we identified 22,068 patients (33.8%) who had undergone laparoscopic appendectomies, according to the ICD-9-CM procedure code 47.01.

### Key Variables of Interest

The key independent variable of interest was whether the performed appendectomy was laparoscopic or open. The key dependent variables included the 30-day re-admission for the treatment of acute appendicitis, the length of stay (LOS), and the cost per discharge (defined as the aggregate monetary value of all the itemized costs of all the services and disposables billed to the NHI).

### Statistical Analysis

We used the SAS package (version 9.1) for statistical analysis in this study. The Pearson χ^2^ tests were used to compare the difference between patients who had undergone laparoscopic appendectomies and patients who had undergone open appendectomies, according to the following characteristics: surgeon (age, sex, and practice location); hospital (teaching status and hospital accreditation level); and patient (age, sex and Charlson Cormobidity Index (CCI) score). Mann-Whitney tests were carried out to examine the relationship between the LOS, the cost per discharge, and the appendectomy method. The generalized estimated equation (GEE) was utilized to explore the difference in 30-day readmission rates between laparoscopic appendectomies and open appendectomies, after accounting for any clustering of the sampled patients among particular surgeons, and adjusting for surgeon, hospital, and patient characteristics. In addition, hierarchical linear regressions were performed to examine the relationship between the use of laparoscopy, the natural logarithm of the LOS, and the cost per discharge.

We also calculated a propensity score for each patient and adjusted for propensity in all regression models. A propensity score was initially used to balance demographic and treatment characteristics, which were distributed unequally between patients who had undergone laparoscopic appendectomies and open appendectomies. Because the probability of undergoing laparoscopic appendectomies depends on the opinion of both the surgeon and the patient, variables for the surgeon’s age, sex, and practice location, as well as the patient’s age, sex and CCI score, were entered into a multivariable logistic regression model as predictors, to calculate the expected probability of undergoing laparoscopic appendectomies for each patient. A two-sided *p* value of less than, or equal to, 0.05 was considered to be statistically significant.

## Results

Table 1 presents the distribution of patient, surgeon, and hospital characteristics, according to the use of laparoscopy. Of the 65,339 patients admitted for the treatment of acute appendicitis between January 2007 and December 2009, the mean age was 36.7 (±19.3); 35.0 for patients who had undergone laparoscopic appendectomies, and 37.7 for patients who had undergone open appendectomies (*p*<0.001). Patients undergoing laparoscopic appendectomies were more likely to have a CCI score of zero than patients undergoing open appendectomies (94.5% versus 91.3%, *p*<0.001). Considering surgeon characteristics, patients with acute appendicitis were more likely to undergo laparoscopic appendectomies by younger surgeons, (*p*<0.001) and surgeons who practiced in urban areas (*p*<0.001) than patients undergoing open appendectomies. In addition, a higher proportion of laparoscopic appendectomies took place in teaching hospitals (*p*<0.001) and medical centers (*p*<0.001).

**Table 1:** Distributions of characteristics of patient, surgeon, and hospital according to the use of laparoscopy (n = 65,339).

Variable	All	Use of Laparoscopy		*p* value
		Yes	No	
**Patient characteristics**				
No. (%) of patients	65,339	22,068	43,271	
Age, mean (SD), years	36.7 (19.3)	35.0 (17.7)	37.7 (20.0)	<0.001
No. (%) of Female	29,930 (45.8)	10,576 (47.9)	19,354 (44.7)	<0.001
Age (years), *n* (%)				<0.001
≤19	13,772 (21.1)	4,819 (21.9)	8,953 (20.7)	
20∼39	25,320 (38.7)	9,257 (41.9)	16,063 (37.1)	
40∼59	17,501 (26.8)	5,805 (24.3)	11,696 (27.0)	
≥60	8,746 (13.4)	2,187 (9.9)	6,559 (15.2)	
Charlson Comorbidity Index score				<0.001
0	60,381 (92.4)	20,864 (94.5)	39,517 (91.3)	
1	3,639 (5.6)	971 (4.4)	2,668 (6.2)	
2	610 (0.9)	104 (0.5)	506 (1.2)	
3 or more	709 (1.1)	129 (0.6)	580 (1.3)	
**Surgeon characteristics**				
No. (%) of surgeons	2,536	414 (16.4)	2,122 (83.6)	
Age, mean (SD), years	43.7 (8.7)	40.9 (7.0)	44.3 (8.8)	<0.001
No. (%) of Female	239 (9.4)	40 (9.6)	199 (9.4)	0.894
Age (years), *n* (%)				<0.001
≤40	1,064 (42.0)	226 (53.6)	838 (39.3)	
41∼50	916 (36.1)	155 (34.5)	761 (33.3)	
≥51	556 (21.9)	43 (11.9)	513 (27.4)	
No. (%) of Practice location				<0.001
Urban	1,849 (72.9)	326 (78.4)	1,523 (71.8)	
Rural	687 (27.1)	88 (21.6)	599 (28.2)	
**Hospital characteristics**Hospital teaching status				<0.001
Yes	2,020 (79.7)	381 (92.0)	1,639 (77.2)	
No	516 (20.3)	37 (8.0)	479 (22.8)	
Hospital level				<0.001
Medical center	837 (33.0)	187 (45.2)	650 (30.7)	
Regional hospital	1,048 (41.3)	183 (44.0)	865 (40.7)	
District hospital	651 (25.7)	44 (10.8)	607 (28.6)	

Table 2 shows the relationship between the use of laparoscopy, 30-day re-admission rate, length of stay, and the cost per discharge. It reveals that a significantly lower proportion of patients undergoing laparoscopic appendectomies had been re-admitted within 30 days of their index appendectomy, in comparison to patients undergoing open appendectomies (0.66% versus 1.925, *p*<0.001). When compared with patients undergoing open appendectomies, patients undergoing laparoscopic appendectomies had a shorter LOS (4.01 versus 5.33 days, *p*<0.001) and a higher cost per discharge (NT$40,554 versus NT$38,509, *p*<0.001. In 2007, the average exchange rate was US$1 = NT$31.0).

**Table 2:** Relationships between 30-day readmission rate, length of stay, cost per discharge, and the use of laparoscopy.

Variable	Method of Appendectomy		
	Laparoscopic	Open	
	N, % or mean (SD)		P value
30-day readmission rate	146 (0.66)	832 (1.92)	<0.001
Length of stay (days)	4.01 (2.90)	5.33 (5.12)	<0.001
Cost per discharge (NT$)	40,554 (23,306)	38,509 (48,941)	<0.001

SD = standard deviation; In 2007, the average exchange rate was US$1 = NT$31.0.

Table 3 illustrates the covariate-adjusted relationship between the use of laparoscopy, 30-day re-admission, the LOS, and the cost per discharge. GEE showed that the odds ratio (OR) of 30-day readmission for patients undergoing laparoscopic appendectomies was 0.38 (95% CI = 0.33–0.46) that of patients undergoing open appendectomies, after adjusting for surgeon, hospital, and patient characteristics, as well as accounting for the clustering effect for any particular surgeons and calculating the propensity score. In addition, after adjusting for other confounding factors, patients undergoing laparoscopic appendectomies experienced a significantly shorter LOS and were charged a higher cost per discharge than patients undergoing open appendectomy.

**Table 3:** Adjusted relationships between 30-day readmission, length of stay, cost per discharge, and the use of laparoscopy.

Variables	30-day readmission	Log (length of stay)	Log (costs)
	OR, 95%CI	Parameter estimate, SE	
Laparoscopy			
Yes	0.38*** (0.33–0.46)	−0.157*** (0.005)	0.213*** (0.005)
No	1.00	1.00	1.00
Surgeon age (years)			
<41	1.00	1.00	1.00
41∼50	1.01 (0.78–1.30)	−0.053*** (0.009)	−0.039*** (0.009)
>50	1.03 (0.70–1.51)	−0.109*** (0.014)	−0.082*** (0.014)
Surgeon gender			
Male	0.51*** (0.36–0.71)	0.035* (0.014)	0.058*** (0.014)
Female	1.00	1.00	1.00
Practice location			
Urban	0.91 (0.78–1.07)	−0.030*** (0.006)	−0.019** (0.006)
Rural	1.00	1.00	1.00
Hospital teaching status			
Yes	0.54*** (0.44–0.66)	−0.011 (0.010)	0.094*** (0.009)
Hospital level			
Medical center	1.00	1.00	1.00
Regional hospital	1.19 (0.97–1.47)	−0.022** (0.007)	−0.096*** (0.007)
District hospital	2.59*** (1.87–3.59)	−0.100*** (0.012)	−0.211*** (0.011)
Patient age (years)			
≤19	1.00	1.00	1.00
20∼39	0.59*** (0.49–0.71)	−0.061*** (0.007)	−0.017 (0.006)
40∼59	0.96 (0.78–1.16)	0.073*** (0.007)	0.060*** (0.007)
≥60	1.46** (1.15–1.85)	0.284*** (0.010)	0.226*** (0.009)
Patient gender			
Male	1.02 (0.89–1.16)	0.024*** (0.005)	0.034*** (0.004)
Female	1.00	1.00	1.00
Charlson Comorbidity Index score			
0	1.00	1.00	1.00
1	1.58*** (1.26–1.98)	0.264*** (0.011)	0.213*** (0.005)
2	2.49*** (1.68–3.70)	0.403*** (0.024)	0.362*** (0.023)
3 or more	2.91*** (2.04–4.15)	0.681*** (0.023)	0.685*** (0.021)
Propensity score	0.31* (0.10–0.89)	0.292*** (0.041)	0.170***(0.039)

Note: SE = standard error,

* p<0.05;

** *p*<0.01;

*** *p*<0.001.

We investigated the adjusted relationship between the use of laparoscopy, 30-day re-admission, the LOS, and the cost per discharge, stratified according to whether the patient had perforated appendicitis (Tables 4 and 5). The results showed that patients undergoing laparoscopic appendectomies had significantly lower odds of 30-day re-admission, a shorter LOS, and a higher cost per discharge than patients undergoing open appendectomy.

**Table 4:** Adjusted relationships between 30-day readmission, length of stay, cost per discharge, and the use of laparoscopy for patients with perforated appendicitis.

Variables	Perforated appendicitis		
	30-day readmission	Log (length of stay)	Log (costs)
	OR, 95%CI	Parameter estimate, SE	
Laparoscopy			
Yes	0.42*** (0.33–0.53)	−0.050*** (0.005)	0.304*** (0.005)
No	1.00	1.00	1.00
Surgeon age (years)			
<41	1.00	1.00	1.00
41∼50	1.12 (0.79–1.58)	−0.044*** (0.009)	−0.018* (0.009)
>50	1.19 (0.71–2.00)	−0.094*** (0.014)	−0.051*** (0.014)
Surgeon gender			
Male	0.56* (0.34–0.90)	0.013*** (0.014)	0.032 (0.014)
Female	1.00	1.00	1.00
Practice location			
Urban	0.90 (0.74–1.10)	−0.036*** (0.006)	−0.024*** (0.006)
Rural	1.00	1.00	1.00
Hospital teaching status			
Yes	0.53*** (0.41–0.68)	−0.052*** (0.009)	0.060*** (0.009)
Hospital level			
Medical center	1.00	1.00	1.00
Regional hospital	1.55** (1.14–2.10)	0.044*** (0.007)	−0.067*** (0.007)
District hospital	3.70*** (2.36–5.81)	−0.002*** (0.012)	−0.157*** (0.012)
Patient age (years)			
≤19	1.00	1.00	1.00
20∼39	0.61*** (0.48–0.77)	0.030*** (0.006)	0.044*** (0.006)
40∼59	0.89 (0.69–1.15)	0105*** (0.007)	0.091*** (0.007)
≥60	1.15 (0.82–1.61)	0.227*** (0.010)	0.163*** (0.009)
Patient gender			
Male	1.06 (0.89–1.26)	−0.009 (0.005)	0.016*** (0.010)
Female	1.00	1.00	1.00
Charlson Comorbidity Index score			
0	1.00	1.00	1.00
1	2.09*** (1.52–2.86)	0.195*** (0.012)	0.109*** (0.011)
2	3.93*** (2.26–6.83)	0.329*** (0.029)	0.280*** (0.028)
3 or more	4.16*** (2.44–7.09)	0.667*** (0.028)	0.541*** (0.026)
Propensity score	0.22 (0.04–1.11)	0.389*** (0.041)	0.114** (0.039)

Note: *B* Parameter estimate, SE standard error,

* p<0.05;

*** *p*<0.001.

**Table 5:** Adjusted relationships between 30-day readmission, length of stay, cost per discharge, and the use of laparoscopy for patients with acute appendicitis (without perforated appendicitis).

Variables	Without perforated appendicitis		
	30-day readmission	Log (length of stay)	Log (costs)
	OR, 95%CI	Parameter estimate, SE	
Laparoscopy			
Yes	0.44*** (0.32–0.59)	−0.229*** (0.011)	0.077*** (0.012)
No	1.00	1.00	1.00
Surgeon age (years)			
<41	1.00	1.00	1.00
41∼50	0.94 (0.65–1.37)	−0.030 (0.018)	−0.064 (0.019)
>50	0.91 (0.51–1.64)	−0.077** (0.028)	−0.116** (0.031)
Surgeon gender			
Male	0.44*** (0.27–0.71)	0.004 (0.028)	0.079* (0.031)
Female	1.00	1.00	1.00
Practice location			
Urban	0.92 (0.72–1.18)	−0.031* (0.012)	−0.018 (0.014)
Rural	1.00	1.00	1.00
Hospital teaching status			
Yes	0.50*** (0.35–0.72)	−0.020 (0.022)	0.114*** (0.024)
Hospital level			
Medical center	1.00	1.00	1.00
Regional hospital	1.03 (0.76–1.38)	−0.079*** (0.013)	−0.103*** (0.015)
District hospital	2.07** (1.26–3.39)	−0.176*** (0.025)	−0.241*** (0.027)
Patient age (years)			
≤19	1.00	1.00	1.00
20∼39	0.68* (0.49–0.94)	−0.121*** (0.014)	−0.090*** (0.015)
40∼59	1.01 (0.74–1.37)	−0.037 (0.015)	−0.038 (0.016)
≥60	1.49* (1.05–2.12)	0.115*** (0.017)	0.131*** (0.016)
Patient gender			
Male	0.89 (0.73–1.09)	0.027** (0.009)	0.024** (0.010)
Female	1.00	1.00	1.00
Charlson Comorbidity Index score			
0	1.00	1.00	1.00
1	1.09 (0.80–1.50)	0.227*** (0.017)	0.272*** (0.018)
2	1.50 (0.85–2.64)	0.313*** (0.034)	0.340*** (0.038)
3 or more	1.88** (1.17–3.01)	0.501*** (0.031)	0.698*** (0.034)
Propensity score	0.40 (0.07–2.20)	0.284*** (0.080)	0.242** (0.088)

Note: *B* Parameter estimate, SE standard error,

* *p*<0.05;

** *p*<0.01;

*** *p*<0.001.

## Discussion

The first report of laparoscopic appendectomy performed in Taiwan was published in 1999 by Yao et al. [9]. Since 2000, laparoscopic appendectomies have become popular in Taiwan because they were demonstrated in comparative studies to be well-tolerated by children, the elderly, and patients with perforated appendicitis [11]–[14]. We analyzed the dataset from January 2007 to December 2009 to investigate the association between laparoscopic appendectomies, 30-day re-admission of acute appendicitis, the LOS, and the cost per discharge. Of the 65,339 patients admitted for the treatment of acute appendicitis, 22,068 (33.8%) patients received laparoscopic surgery. Patients in the laparoscopic group were more likely to be younger and female. Our data reflect the penetration rate more precisely than that of previous reports [15].

There were more than 2,500 surgeons in Taiwan performing appendectomies during the study period, but only 414 (16.4%) surgeons performed appendectomies using the laparoscopic approach, of whom the majority (near 90%) were younger than 50 years old. Historically, most of the surgeons performing laparoscopic appendectomies in Taiwan received laparoscopic training before the age of 40. The situation is similar in the U.S., where surgeons recertifying after 10 years performed more laparoscopic procedures compared with those recertifying after 20 or 30 years [16].

In the present study, more than 90% of laparoscopic surgeries were performed in teaching hospitals, which may be accounted for by the role of appendectomies in laparoscopic training [17], [18]. In Canada, less than 25% of appendectomies were performed with laparoscopy in more than half (53%) of teaching (University and Affiliated) hospitals [19]. However, we did not analyze the penetration rate of laparoscopic appendectomies among various hospitals. Although Taiwan’s healthcare system effectively reduced the disparities between rural and urban access to healthcare, with the ultimate outcome of health [20], the rate of laparoscopic appendectomy was significantly lower in rural areas than urban areas.

Consistent with previous studies [4], [6], [8], we identified a shorter LOS and significantly higher procedural cost in the laparoscopic group, compared with the open appendectomy group. Although the cost was significantly higher in the laparoscopic group, only a 5% increase was noted, which is lower than previously reported [8]. One possible explanation for the lower cost in the laparoscopic group is that more expensive instruments, such as endo-GIA and harmonic scalpels, are seldom used for laparoscopic appendectomies in Taiwan.

Post-operative intra-abdominal abscesses remain a major concern with laparoscopic appendectomies, especially in cases of complicated appendicitis, as intra-abdominal abscesses are associated with the need for consequent interventions and re-admissions [7]. In the current study, the 30-day readmission rate was much lower in patients who had undergone laparoscopic appendectomies (0.66% versus 1.925, *p*<0.001). In comparison with open appendectomies, the adjusted OR of the 30-day re-admission rate for patients with laparoscopic appendectomies was 0.38 (95% CI = 0.33 to 0.46). For complicated appendicitis, previous studies demonstrated that laparoscopic appendectomies increased the risk for intra-abdominal abscess and consequent re-admission [5], [7]. In the present study, the odds ratio for the 30-day re-admission rate and the LOS all favored the laparoscopic approach for both simple and complicated appendicitis. The benefit of a reduced LOS might be more prominent for patients older than 65 years, patients with co-morbidities, and patients with complicated appendicitis [15].

The strengths of this study include the use of a nationwide population-based dataset and the availability of an adequate study period for analysis. No learning curve problem was present regarding the laparoscopic techniques used for the appendectomies analyzed in this study. However, this study has some limitations. First, the dataset used does not contain data on post-operative course, such as the time to first flatus passage, time to oral intake, or intensity of pain. Second, the operation time and the impact of minor complications, such as superficial wound infections, were not studied. However, the LOS can represent the general condition of the post-operative course, and the cost of admission is influenced by operation time. Huang and Wei reported that superficial wound infections were less severe and easier to treat in laparoscopic groups than in open groups [11].

In conclusion, this study found that laparoscopic appendectomies had a lower 30-day re-admission rate and a shorter LOS, but a slightly higher cost per discharge than open appendectomies. Laparoscopic appendectomies should be the treatment of choice for both simple and complicated appendectomy, as the slightly increased cost of admission can be balanced by reducing the rate of re-admission.
